# Durvalumab-Induced Myocarditis and Dilated Cardiomyopathy in a Patient With Non-small Cell Lung Cancer: A Diagnostic Conundrum

**DOI:** 10.7759/cureus.51456

**Published:** 2024-01-01

**Authors:** Ali Khreisat, Nathanial Bartosek, Tanya Amal, Bhavinkumar Dalal

**Affiliations:** 1 Internal Medicine, Beaumont Hospital, Royal Oak, USA; 2 Pulmonary Critical Care Medicine, Corewell Health William Beaumont Hospital, Royal Oak, USA

**Keywords:** durvalumab, non-small cell lung carcinoma (nsclc), nonischemic cardiomyopathy, dilated cardiomyopthy, myocarditis, immune-checkpoint inhibitors

## Abstract

Immune checkpoint inhibitors have been a therapeutic oncological breakthrough in managing diverse malignancies. We present a 78-year-old male with stage IIIb non-small cell lung cancer (NSCLC) managed by concurrent chemotherapy with carboplatin/pemetrexed and radiotherapy followed by monthly durvalumab injections. He presented to the hospital with shortness of breath and fluid overload after eight months of starting durvalumab. Workup, including laboratory investigations, coronary angiography, and stress myocardial magnetic resonance imaging, increased our suspicion for the diagnosis of durvalumab-induced myocarditis and nonischemic dilated cardiomyopathy. He was managed with aggressive diuresis and pulse dose steroids with an improvement in his symptoms and his cardiac function. This case illustrates an under-reported clinical side effect in the era of advancement in oncological immunotherapy.

## Introduction

Durvalumab, a type of immune checkpoint inhibitor (ICI), is a monoclonal antibody that targets and blocks the interaction between program death-1 ligand (PD1-L) and program death receptor 1 (PD-1), improving the patient's immune surveillance of cancer cells and therefore creating an anticancer immunoreaction through enhanced T-cell response [1,2]. First approved for urothelial carcinoma in 2017, durvalumab is now indicated for stage III non-small cell lung cancer (NSCLC), which is not amenable to surgical resection, extensive-stage small cell lung cancer, and advanced/metastatic biliary tract cancer [3]. As an ICI, durvalumab causes immune-mediate adverse events (imAEs), pneumonitis, thyroid disorders, and dermatitis/rash, with diarrhea/colitis being the most common [4,5]. Rarely, durvalumab can be associated with cardiac imAEs, including myocarditis, pericarditis, arrhythmias, and coronary vasculitis [6]. As per Mahmood SS's multicenteric observational study, the estimated incidence of ICI-myocarditis is 1.14% [7]. Our case outlines myocarditis and dilated cardiomyopathy as a rare cardiac adverse event of durvalumab.

## Case presentation

A 78-year-old caucasian male with a past medical history of hypertension, obstructive sleep apnea, coronary artery disease status post-drug-eluting stent placement to the right coronary artery 10 years prior to presentation, remote 35-pack-year smoking history, and stage IIIb lung adenocarcinoma diagnosed eight months before his hospital admission on concurrent chemoradiotherapy and monthly maintenance durvalumab injections. He was not a candidate for surgical resection due to mediastinal lymph node metastasis.

He presented to the emergency department with dyspnea on exertion, productive cough, and progressive bilateral lower limb edema of one-week duration. He was placed on a 2-liter nasal cannula for hypoxia; however, his other vital signs were stable. Physical examination showed jugular venous distension, lower limb pitting edema, and bilateral inspiratory crackles. His daily home medications include aspirin 81 mg and rosuvastatin 40 mg. Laboratory investigations showed elevated cardiac biomarkers, including brain natriuretic peptide (BNP) of 1783 pg/ml and high-sensitivity troponin I of 106 ng/L that peaked at 123 ng/L (normal level is <=35 ng/L). An electrocardiogram (ECG) showed sinus tachycardia at 110 beats/minute without T-wave or ST segment changes. Cardiomegaly and pulmonary vascular congestion were noted on the chest X-ray. A two-dimensional transthoracic echocardiogram showed a mildly reduced ejection fraction of 40% and global hypokinesis (Video 1). One month before starting immunotherapy, his baseline screening transthoracic echocardiogram showed an ejection fraction of 65% with no regional wall motion abnormalities.

**Video 1:** 2D transthoracic echocardiogram showing an ejection fraction of 40% and global hypokinesis. No pericardial effusion or severe valvular heart disease was noted.

**Figure 1:**
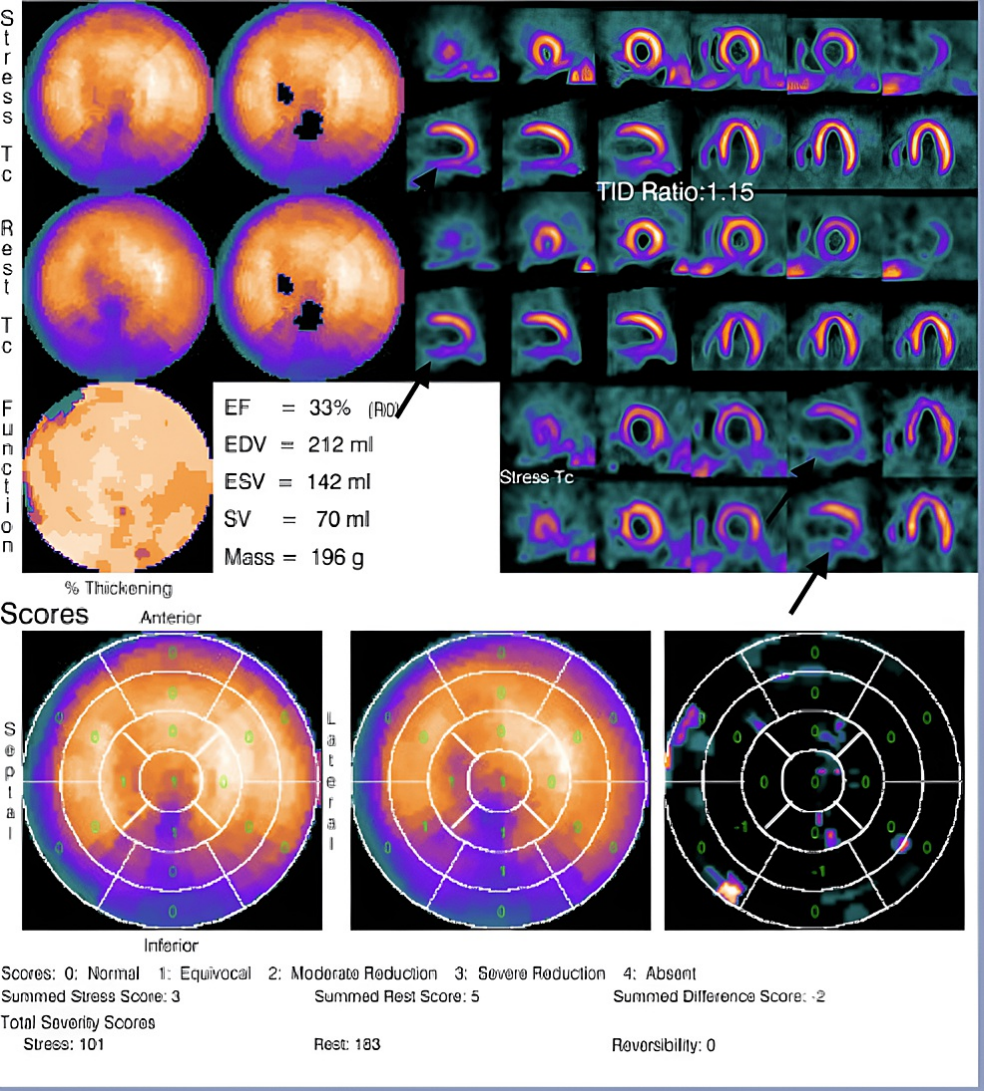
Myocardial perfusion imaging shows persistent moderate fixed perfusion and wall motion defect in the inferior and basal walls without reversibility (arrows), representing 16% of the total left ventricular myocardium

The patient was treated with three days of intravenous furosemide, which improved his oxygen requirements, shortness of breath, and lower limb edema. Repeat BNP trended down to 410 pg/ml. To rule out ischemic etiologies of his new cardiomyopathy, he underwent a left heart catheterization that showed 70% narrowing of the proximal left anterior descending (LAD) with a fractional flow reserve (FFR) of 0.78. He historically denied exertional chest pain. Given the unusual nature of his positive FFR and the lack of anginal symptoms, regadenoson myocardial perfusion imaging (MPI) was performed. It showed fixed perfusion defect in the inferior and basal myocardial walls and no ischemia in the LAD territory (Figure 1).

**Figure 2:**
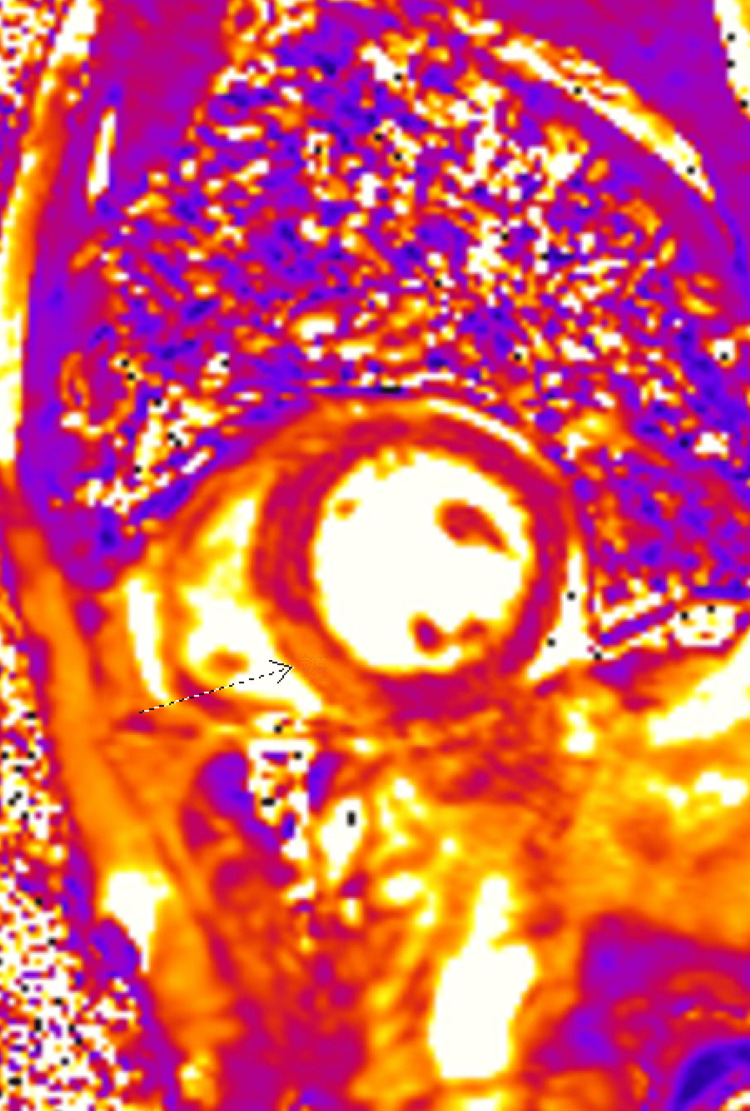
Stress cardiac MRI showing late gadolinium enhancement in the basal inferior and inferolateral wall (arrow) suggestive of myocarditis

Stress cardiac magnetic resonance imaging (CMRI) was performed to investigate nonischemic etiologies for his new cardiomyopathy, and it showed late gadolinium mid-myocardial enhancement in the basal and inferolateral wall, likely due to inflammation and edema. There was no stress-induced ischemia (Figure 2). These findings were highly suggestive of myocarditis. Due to the lack of ischemia and findings of myocarditis on cardiac MRI, the decision was made to forgo percutaneous coronary intervention to the LAD stenosis. Infectious workup was negative, including respiratory viral panel, syphilis, human immunodeficiency virus (HIV), echovirus, and coxsackievirus b antibody testing. Angiotensin-converting enzyme and ferritin levels were within normal range. Suspicion was raised for immune checkpoint inhibitor-induced myocarditis since other possible etiologies for myocarditis were excluded. An endomyocardial biopsy was not performed due to patient refusal.

He was started on pulse-dose steroid therapy with one gram of intravenous methylprednisolone for three days, followed by a tapering course of prednisone for a total duration of six weeks. He was also started on guideline-directed medical therapy (GDMT) with sacubitril/valsartan 49/51 mg twice daily and metoprolol succinate 25 mg daily. Two months after his hospital discharge, he was evaluated in the outpatient cardiology office, with significant symptomatic improvement in his shortness of breath along with trace lower limb edema. Follow-up transthoracic echocardiogram showed improvement in the ejection fraction to 60%. Durvalumab was held from his future cancer treatment, and he was continued on concurrent chemoradiation therapy with pemetrexed plus carboplatin.

## Discussion

Immune checkpoint inhibitors (ICIs) have ushered in a new era in cancer therapy, offering substantial benefits in managing various malignancies. Our case report highlights a rare and underreported cardiac adverse event associated with durvalumab, an immune checkpoint inhibitor used in the treatment of stage IIIb non-small cell lung cancer (NSCLC). The patient, in this case, presented with symptoms of heart failure and was subsequently diagnosed with durvalumab-induced myocarditis and nonischemic dilated cardiomyopathy. Myocarditis typically presents within the first one to two months after the initiation of ICI, but our case, along with others, highlights the possibility of delayed onset [8]. Myocarditis occurred after eight months of durvalumab therapy in our patient. This contrasts with the general trend observed in the literature, where most cases of ICI-related myocarditis present within the first one to two months after the initiation of treatment [9]. This prolonged latency period underscores the importance of maintaining vigilance for cardiac adverse events throughout the course of ICI therapy, necessitating ongoing surveillance and early intervention.

The diagnosis of durvalumab-induced myocarditis and nonischemic dilated cardiomyopathy was established through a comprehensive workup, including laboratory investigations, coronary angiography, and stress myocardial magnetic resonance imaging (MRI). Notably, the use of MRI allowed for a definitive diagnosis, differentiating it from other potential causes and confirming the cardiac involvement associated with ICI therapy. Endomyocardial biopsy remains the gold standard test to diagnose myocarditis. Steroid responsiveness played a crucial role in managing this case, highlighting the necessity of distinguishing immune-related myocarditis from infectious causes. Steroid therapy not only improved the patient's symptoms but also contributed to the recovery of myocardial function. This aligns with the broader literature, emphasizing the effectiveness of steroids in reducing major adverse cardiovascular events associated with ICI-related myocarditis [10].

The pathophysiology of ICI-related myocarditis involves the dysregulation of immune responses. PDL-1 is expressed in various cell types, including tumor cells, T cells, endothelial cells, and epithelial cells, and is crucial in protecting cells from T-cell-mediated immune attacks. The upregulation of PD-L1 on cardiac endothelial cells depends on T-cell-derived interferon-γ, suggesting a complex interplay in the immune response [11]. Also, T cells predominate the tumor environment in NSCLC [12]. Apart from T-cells, secondary activation of polymorphonuclear leukocytes also adds to myocardial damage; these pathomechanisms are in line with the effectiveness of corticosteroids in decreasing the immune response. Furthermore, the murine model studies highlighted the intricate relationship between PD-L1 signaling, T-cell-derived interferon-γ, and the subsequent development of myocarditis. This provides valuable insights into the mechanical aspects of ICI-related cardiotoxicity and emphasizes the need for a nuanced understanding of the immune responses involved [13].

In our patient, the diagnosis of durvalumab-induced myocarditis was confirmed by the exclusion of other etiologies of myocarditis and dilated cardiomyopathy, such as viral myocarditis, ischemia, and infiltrative cardiac disorders, the improvement of our patient's cardiac function by stopping durvalumab and starting high-dose steroids. Also, the characteristic cardiac MRI findings of patchy myocardial late gadolinium enhancement were highly suggestive of ICI-induced myocarditis. This suggests that a thorough laboratory workup guided by cardiac MRI findings can circumvent the use of more invasive procedures like cardiac biopsy.

In conclusion, our case contributes to the growing body of evidence highlighting the diverse and unpredictable nature of ICI-related cardiac adverse events. The delayed onset observed in this case underscores the necessity for ongoing surveillance, and the successful management with steroids highlights the importance of early intervention. A comprehensive understanding of the pathophysiology is crucial for timely diagnosis and appropriate management, ensuring the delicate balance between the benefits and risks of ICI therapy in the oncological setting.

## Conclusions

Rare cases of ICI-related adverse events like myocarditis are yet to be evaluated. Our case adds to the growing body of evidence suggesting that ICI-related myocarditis can occur beyond the commonly reported timeframe. Understanding the nuances of the immune response, especially the role of PD-L1 and interferon-γ, is crucial for unraveling the complexities of this adverse event. Vigilance, early detection, and prompt management, often with corticosteroids, remain pivotal in mitigating the potentially life-threatening consequences of ICI-related myocarditis.
